# Case series and review of Ayurvedic medication induced liver injury

**DOI:** 10.1186/s12906-021-03251-z

**Published:** 2021-03-13

**Authors:** Christopher M. Karousatos, Justin K. Lee, David R. Braxton, Tse-Ling Fong

**Affiliations:** 1 Keck School of Medicine, University of Southern California, Los Angeles, CA USA; 2 Hoag Memorial Hospital Presbyterian, Newport Beach, CA USA; 3 Division of Gastrointestinal and Liver Diseases, Keck School of Medicine, University of Southern California, 1510 San Pablo Street, 2/F, Los Angeles, CA 90033 USA

**Keywords:** Ayurveda, Ayurvedic medicine, Drug induced liver injury (DILI), Herb induced liver injury (HILI), Traditional Chinese medicine, Roussel Uclaf causality assessment method

## Abstract

**Background:**

Complementary and alternative medicine use among Americans is prevalent. Originating in India, Ayurvedic medicine use in the United States has grown 57% since 2002. CAM accounts for a significant proportion of drug induced liver injury in India and China, but there have been only three reports of drug induced liver injury from Ayurvedic medications in the U.S. We report three cases of suspected Ayurvedic medication associated liver injury seen at a Southern California community hospital and review literature of Ayurvedic medication induced liver injury.

**Case presentations:**

Three patients presented with acute hepatocellular injury and jaundice after taking Ayurvedic supplements for 90–120 days. First patient took Giloy Kwath consisting solely of *Tinospora cordifolia.* Second patient took Manjishthadi Kwatham and Aragwadhi Kwatham, which contained 52 and 10 individual plant extracts, respectively. Third patient took Kanchnar Guggulu, containing 10 individual plant extracts. Aminotransferase activities decreased 50% in < 30 days and all 3 patients made a full recovery. Roussel Uclaf Causality Assessment Method (RUCAM) scores were 7–8, indicating probable causality. These products all contained ingredients in other Ayurvedic and traditional Chinese medicines with previously reported associations with drug induced liver injury.

**Conclusions:**

These patients highlight the risk of drug induced liver injury from Ayurvedic medications and the complexity of determining causality. There is a need for a platform like LiverTox.gov to catalog Ayurvedic ingredients causing liver damage.

**Supplementary Information:**

The online version contains supplementary material available at 10.1186/s12906-021-03251-z.

## Background

Originating in India thousands of years ago, Ayurveda is among the oldest healing systems in existence, with a focus on harmonious living and self-sustainability [1, 2]. Diseases are viewed in the context of their effects on an individual’s dosha or mind-body type with respect to energies of the five elements: earth, water, fire, air, and ether [3]. Though illness prevention is promoted through lifestyle modifications, medicinal herbs are an integral part of Ayurveda [4]. Ayurveda is prominent in India, with the government recognizing and funding the practice, development, and research of Ayurvedic Medicine (AM) [5]. The Ayurvedic sector in India had an estimated market value of three billion USD in 2016 and its market value is expected to continue growing [6].

Though limited data exists quantifying the money spent on AM in the United States, recent trends suggest rising popularity [7]. Currently, the most popular Ayurvedic supplement in the United States is Ashwagandha (*Withania somnifera*), with sales in 2018 totaling over 7 million dollars, an increase of 165.9% from the year prior, according to a market report published by the American Botanical Council [8]. More broadly, the National Center for Health Statistics survey on Complementary and Alternative Medicine (CAM) in 2015 showed that 241,000 American adults used AM, a nearly 57% increase from 2002 [9]. A survey of South Asian Americans in Northern California estimated that about 59% had used or were currently using AM but only 18% mentioned this to their healthcare providers [10]. Given these indicators of rising popularity, awareness of AM and its potential dangers is increasingly important for physicians in the United States.

CAMs make up a larger percentage of drug-induced liver injury (DILI) in Asia where CAM is more prevalent [11, 12]. One study of cirrhotic patients in India reported that of 1666 patients with cirrhosis, 35.7% had acute-on-chronic liver failure secondary to CAM-related DILI on presentation [11]. Like AM, in China, traditional Chinese medicine (TCM) is officially state-supported and institutionalized [13]. A survey of cancer patients of a large urban hospital in China revealed that more than 80% of patients were using TCM in conjunction with Western medicine [14]. In a series of 1985 cases of DILI in China, TCM was implicated in 28% of cases and an additional 28% of cases where TCM was taken in conjunction with Western medication [15]. In contrast, herbal and dietary supplements accounted for only 15.5% of liver injury among cases recorded by the Drug Induced Liver Injury Network in the U.S. [16]. There have been only three cases where specific Ayurvedic medications have been linked to liver damage in the U. S [17, 18] and seven in Europe [18–22]. Other case reports outside of India have come from Canada [23], South America [24], and Israel [25].

Within a 3 month-span, three cases of suspected AM-induced liver injury were seen at a Southern California community hospital. Using the updated Roussel Uclaf Causality Assessment Method (RUCAM), we report these three patients and review the current literature of AM-associated DILI [26]. There are many plant species that are indigenous to India and China due to the proximities of these two countries. To highlight the common ingredients of AM and TCM, the herbal/plant ingredients of the AM supplements taken by these three patients were also cross-referenced with components of TCM that have been reported to be associated with liver injury. This report highlights the substantial challenges in assessing causality in cases of herbal product-induced liver injury.

## Case presentation

**Fig. 1:**
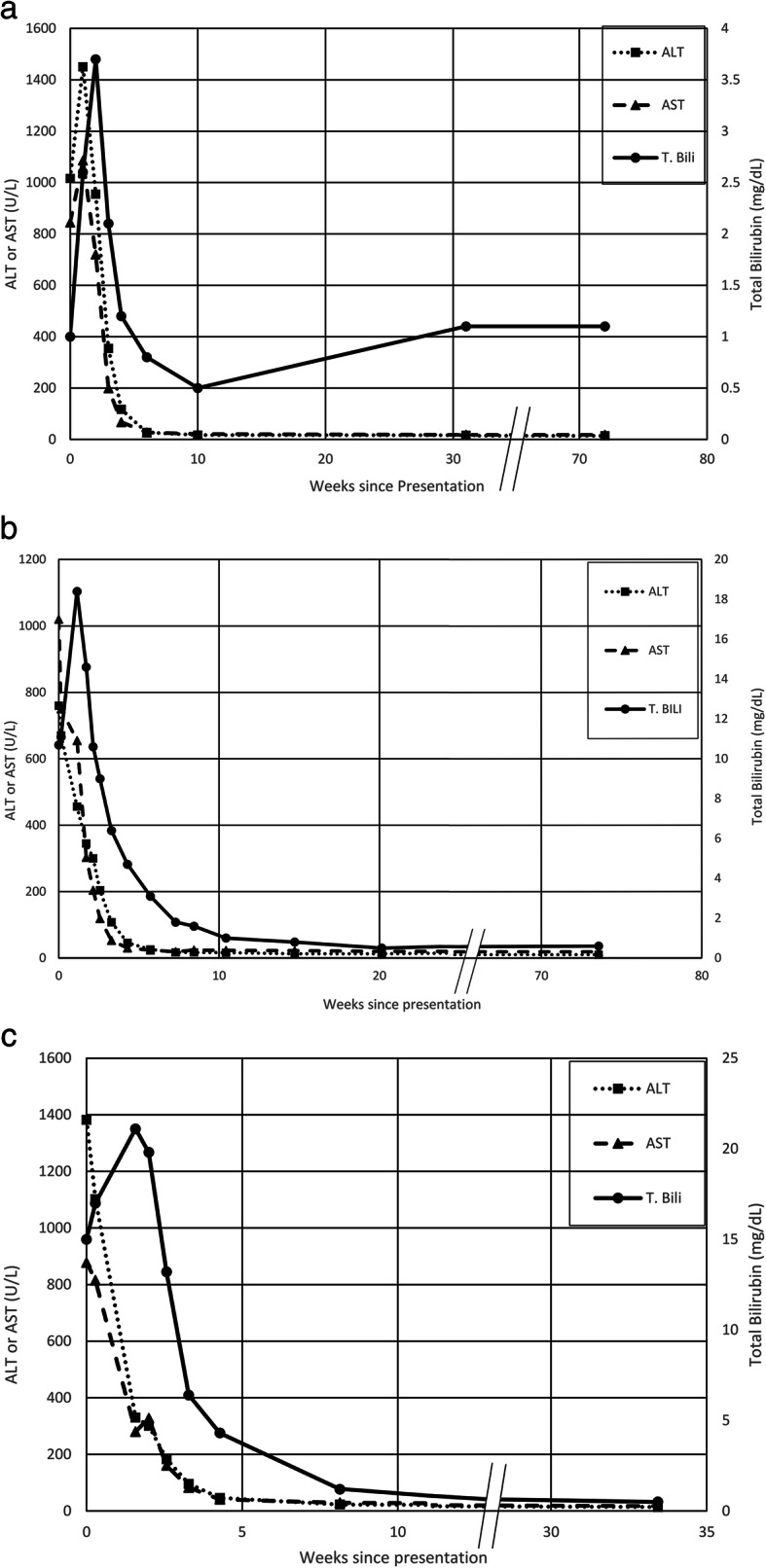
(**a**, **b**, **c**) Alanine aminotransferase activity and bilirubin level of 3 patients

Patient 1 is a 68-year-old South Asian female with a history of hypothyroidism, dyslipidemia and borderline diabetes mellitus who began taking an Ayurvedic supplement, Giloy Kwath, to improve her overall health. Her baseline blood work revealed mildly elevated alanine aminotransferase (ALT) 35 U/L and was otherwise normal. Four months later, routine follow-up blood work revealed acute hepatocellular injury: alkaline phosphatase (AP) 113 U/L, total protein (TP) 6.6 mg, albumin (alb) 4.1 g/dL, total bilirubin (t bili) 1.0 mg/dL, ALT 1016 U/L, aspartate aminotransferase (AST) 844 U/L, and international normalizing ratio (INR) 1.0. Viral hepatitis A, B, C and E serologies were negative. Anti-nuclear antibody (ANA) was weakly positive but anti-smooth muscle antibody (SMA) and anti-liver kidney microsomal antibody (LKM) were negative. Her physical exam was normal. She was asymptomatic until 1 week later when she became jaundiced. At this time, she immediately stopped the supplement. Within 1 month of stopping, her liver tests became normal and remain normal 1 year later (Fig. 1a).

**Fig. 2:**
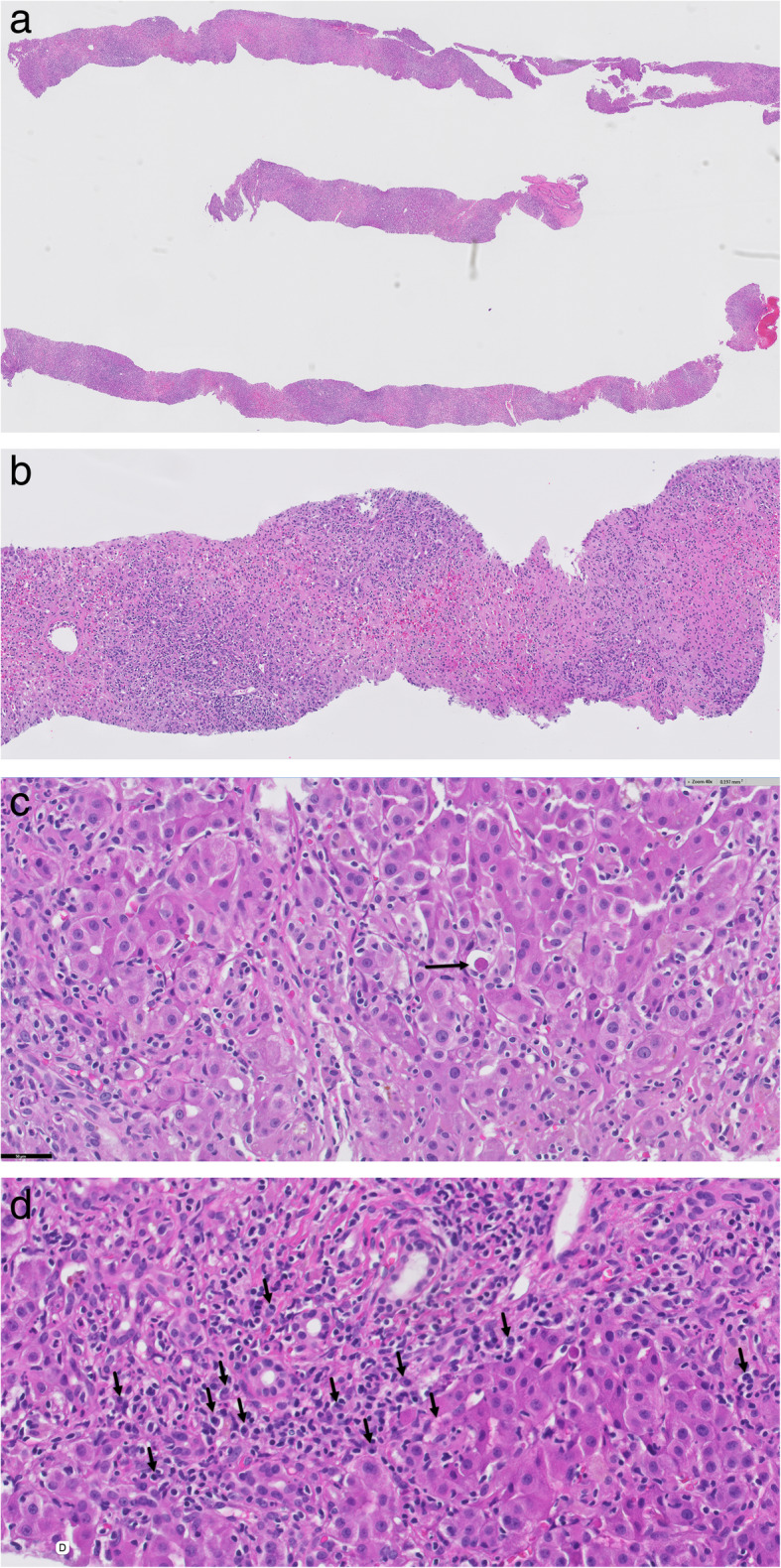
Histopathology showing Severely active panlobular hepatitis with panacinar necrosis and lymphoplasmacytic infiltrates: **a**. Low power (2x Magnification) The portal tracts are expanded at low power with diffuse inflammatory infiltration. **b**. Collapse of hepatocyte lobules illustrate the presence of panacinar necrosis. Note the remnants of the portal tracts marked by the presence of biliary ductules. (Magnification 10x). **c**. Remnant hepatic lobule showing infiltration by predominantly lymphocytes. Apoptotic hepatocytes were conspicuous (arrow). (Magnification 40x). **d**. Numerous plasma cells (arrows) are present in portal tracts showing interface activity. (Magnification 40x)

Patient 2 is a 38-year-old South Asian female with a history of hypothyroidism who began taking Manjishthadi Kwatham and Aragwadhi Kwatham for a skin rash. For the preceding 4 years prior to taking AM, her liver tests were normal. Four months after starting AM, she was initially evaluated for jaundice, fatigue, anorexia, and right upper quadrant discomfort. Her physical exam was significant for marked scleral icterus. Abdominal exam was unremarkable, without hepatomegaly. Blood work on admission revealed AP 175 U/L, TP 7.5 mg, alb 3.7 g/dL, t bili 10.7 mg/dL, ALT 760 U/L, AST 1020 U/L, lactate dehydrogenase (LDH) 923 U/L, INR 1.3, and IgG immunoglobulin 1238 mg/dL. Abdominal ultrasound revealed normal liver echogenicity with a smooth contour. MRI/MRCP confirmed a normal biliary tree. Her gallbladder wall was thickened, measuring 13 mm. Viral hepatitis A, B, C and E serologies were negative. Epstein-Barr IgM antibody was negative. Autoimmune serologies including ANA, SMA, and LKM were negative. Liver biopsy showed severely active pan-lobular hepatitis with bridging necrosis and lymphoplasmacytic infiltrates. There was no definitive fibrosis (Fig. 2). She was treated with prednisone empirically for 6 weeks. Her liver tests normalized 6 weeks after presentation and have remained persistently normal 16 months after discontinuation of corticosteroids (Fig. 1b).

Patient 3 is a 46-year-old Hispanic female who is obese with menometrorrhagia who began taking Kanchnar Guggulu for her menses. Baseline liver tests taken 4 months prior to taking Kanchnar Guggulu were normal. Three months later, she became jaundiced with pruritus and a maculopapular rash. Her abdomen was non-tender and without hepatosplenomegaly. The remainder of her physical exam was unremarkable. Her laboratory studies revealed AP 140 U/L, TP 6.4 mg, alb 4.1 g/dL, t bili 15.0 mg/dL, ALT 1382 U/L, AST 878 U/L, and INR 1.1. Viral hepatitis A, B, C and E serologies were negative. ANA was weakly positive but SMA and LKM were negative. Ultrasound showed a normal size liver with normal echogenicity. There were no gallstones or biliary dilatation. The supplement was discontinued and after 8 weeks her liver tests returned to normal. They remain normal 6 months later (Fig. 1c).

Summary of the clinical characteristics are shown in Table 1 and the compilation of RUCAM for the Ayurvedic supplements are shown in Table 2.

**Table 1:** Clinical characteristics

Patient Characteristics			
	Patient 1	Patient 2	Patient 3
Age (years)	68	38	46
Gender	F	F	F
Ethnicity	Asian	Asian	Hispanic
Herbal Product	Giloy Kwath	Manjishthadi Kwatham Aragwadhadi Kwatham	Kanchnar Guggulu
Time to onset (days)	~ 90	~ 120	~ 90
Peak bilirubin (mg/dL)	3.7	18.4	21.1
Peak alkaline phosphatase (U/L)	200	175	140
Peak ALT (U/L)	1451	760	1382
Peak AST (U/L)	1086	1020	876
R index	21.8	13	29.6
Peak INR	1.0	1.8	1.1
Presentation to peak ALT (days)	8	0	0

**Table 2:** RUCAM for liver injury

	Possible Score	Patient 1	Patient 2	Patient 3
Time to onset	+ 2	+ 2	+ 1	+ 2
5–90 days				
< 5 or > 90 days	+ 1			
Course of ALT after cessation of herb		+ 2	+ 3	+ 2
Decrease > 50% within 8 days	+ 3			
Decrease > 50% within 30 days	+ 2			
Risk factors				
Alcohol +	−1			
Alcohol -	0	0	0	0
Age > 55 years	+ 1	+ 1		
Age < 55 years	0		0	0
No concomitant drugs/herbs		0	0	0
All causes-group I and II-ruled out		+ 2	+ 2	+ 2
Previous hepatotoxicity		+ 1	+ 1	+ 1
Reaction labeled in the product characteristics	+ 2			
Reaction published but not labeled	+ 1			
Response to unintentional re-exposure		0	0	0
Total score for the case		+ 8	+ 7	+ 7

RUCAM score causality levels < 0 excluded causality; 1–2 unlikely; 3–5, possible; 6–8, probable; and > 9, highly probable (ref [26])

All three patients took Ayurvedic medications in quantities as recommended, which contained plant ingredients that have been previously associated with liver injury in a large cases series [27] and case reports of AM-associated liver injury [17–25, 28] (Table 3). Patient 1 took Giloy Kwath that contained a single ingredient, *Tinospora cordifolia*, that was a component in eight preparations of Ayurvedic medications associated with liver injury reported in a large single center case series [27]. Patient 2 took 2 ayurvedic preparations; Manjishthadi Kwatham and Aragwadhadi Kwatham that contained 52 and 10 individual plant extracts, respectively (Table 3). Twenty-three extracts found in Manjishthadi Kwatham were associated with liver injury in prior literature, including *Psoralea cordyfolia* which was reported in two cases where this herb was the lone ingredient in the supplements taken by the patients [20, 21]. In addition to *Tinospora cordifolia*, which was also present in Giloy Kwath taken by Patient 1 and Manjishthadi Kwatham also taken by Patient 2, there were 8 other plant extracts in Aragwadhadi Kwatham associated with liver injury reported in prior literature, detailed in Table 3. Patient 3 took Kanchnar Guggulu, one of the 3 supplements taken by a patient with DILI reported by Dalal et al. [29]. Nine ingredients were found in other preparations associated with DILI described in other case reports or reviews: *Cinnamomum tamala* [17, 27], *Cinnamomum zeylanicum* [27], *Elettaria cardamomum* [17, 27], *Emblica officinalis* [27], *Piper longum* [23, 27], *Piper nigrum* [27], *Terminalia belerica* [17, 23, 27], *Terminalia chebula* [22], *Zingiber officinale* [19, 27].

**Table 3:** Ingredients of Ayurvedic supplements

Patient 1	Patient 2		Patient 3
Giloy Kwath	Manjishthadi Kwatham	Aragwadhadi Kwatham	Kanchnar Guggulu [29]
*Tinospora cordifolia*^a^ [17, 23, 27]	*Acacia catechu*^a^ [17, 20]	*Alstonia scholaris*	*Bauhinia variegata*^a^ [29]
	*Acorus calamus*^a^ [27]	*Andrographis paniculate*^a^ [27]	*Cinnamomum tamala*^a^ [17, 27, 29]
	*Azadirachta indica*^a^ [23, 27, 30]	*Azadirachta indica*^a^ [23, 27, 30]	*Cinnamomum verum*^a^ [17, 29]
	*Cassia fistula*	*Bridelia stipularis*	*Commiphora wightii*
	*Cedrus deodara*^a^ [17, 29]	*Butea monosperma*	*Crataeva nurvala*^a^ [29]
	*Chonemorpha fragrans*	*Calycopteris floribunda*	*Elettaria cardamomum*^a^ [17, 27, 29]
	*Citrullus colocynthis*	*Cassia fistula*	*Embelica officinalis*^a^ [23, 27, 29]
	*Clerodendrum serratum*^a^ [17]	*Cyclea peltata*^a^ [27]	*Piper longum*^a^ [17, 23, 27, 29]
	*Crataeva magna*	*Holarrhena antidysenterica*^a^ [23, 29]	*Piper nigrum*^a^ [27, 29]
	*Cullen corylifolium*	*Marsdenia tenacissima*^a^ [27]	*Terminalia belerica*^a^ [29]
	*Curcuma longa*^a^ [27, 29]	*Monringa oleifera*	*Terminalia chebula*^a^ [23, 27, 29]
	*Cyclea peltate*^a^ [27]	*Plumbago zeylanica*^a^ [17, 27, 29]	*Zingiber officinale*^a^ [19, 20, 27]
	*Cyperus rotundus*^a^ [17, 29]	*Pongamia glabra*	
	*Eclipta prostrata*	*Stereospermuum suaveolens*^a^ [27]	
	*Embelia ribes*^a^ [23, 27, 29]	*Strobilanthes ciliatus*	
	*Embelica officinalis*^a^ [23, 27, 29]	*Tephrosia purpurea*^a^ [23]	
	*Gentiana kurroo*	*Tinospora cordifolia*^a^ [17, 23, 27]	
	*Hemidesmus indicus*^a^ [27]	*Trichosanthes cucumerina*	
	*Holarrhena pubescens*	*Trichosanthes tricuspidata*	
	*Holoptelia integrifolia*		
	*Ichnocarpus frutescens*		
	*Justicia beddomei*		
	*Neopicrorhiza scrophulariiflora*		
	*Oldenlandia corymbosa*		
	*Operculina turpethum*^a^ [17, 27, 29]		
	*Pavoni odorata*		
	*Psoralea corylifolia*^a^ [20, 21]		
	*Phyllanthus emblica*^a^ [17, 27]		
	*Piper longum*^a^ [17, 23, 27, 29]		
	*Plectranthus vettiveroides*		
	*Pterocarpus marsupium*^a^ [17]		
	*Rubia cordifolia*^a^ [17]		
	*Santalum album*^a^ [17]		
	*Streblus asper*		
	*Swertia chirayita*		
	*Tamarindus indica*		
	*Terminalia belerica*^a^ [27]		
	*Terminalia chebula*^a^ [22, 27, 29]		
	*Tinospora cordifolia*^a^ [17, 23, 27]		
	*Tragia involucrata*^a^ [17, 27]		
	*Trichosanthes cucumerina*		
	*Zingiber officinale*^a^ [19, 20, 27]		

^a^ refers to herbs found to be a component of a compound previously associated with AM-induced liver injury

The individual ingredients of these AM supplements were cross-referenced with components of TCMs that have been reported to be associated with liver injury (Table 4). Although *Tinospora cordifolia* (contained in Gilroy Kvath taken by patient 1 and in Manjishthadi Kwatham taken by patient 2) was not reported among TCM, a closely related plant species, *Tinospora crispa*, is contained in Bo Ye Qing Niu Dan which has been associated with liver injury [31, 32]. *Azadirachta indica* contained in Manjishthadi Kwatham is found in Ku Lian Zi as well as *Cinnamomum tamala* (Indian bay leaf) that is contained in Sairei To, which has been reported to cause DILI in TCM supplements [31–33].

**Table 4:** Ingredients common in AM and TCM associated liver injury

AM	Herb	TCM [31]
Bakuchi tablets [20]	*Psoralea corylifolia*	Bai Shi Wan
Babchi Seeds [21]		Shou Wu Pian
Avalguja^a^		Boh Gol Zhee
Liquorice [19]	*Glycyrrhiza glabra*	Bofu Tsu Sho San
		Bai Shi Wan
		Long Dan Xie Gan Tang
		Sairei To
		Da Chai Hu Tang
		Xiao Chai Hu Tang
		Gan Cao
Kaishore (Kishore) Guggal [27]	*Zingiber officinale*	Bofu Tsu Sho San
Pushpadhanva Ras [27]		Da Chai Hu Tang
Bhavana Dravya [27]		Kamishoyosan
Sunthi^a^ [27]		Sairei To
Sonth [27]		Xiao Chai Hu Tang
Kaisoragulu vatika [27]		
Mahayograj Guggul [27]		
Shunthi^a^		
Kaishore (Kishore) Guggal [27]	*Piper nigrum*	Shen Min
Bhavana Dravya [27]		
Maricha^a^		
Pushpadhanva Ras [27]	*Cinnamomum verum*	Sairei To
Tej Patra^a^		
Tvak^a^		
Pushpadhanva Ras [27]	*Cinnamomum tamala*	Sairei To
Tej Patra^a^		
Tvak^a^		
Arishta^a^	Azadirachta indica	Ku Lian Zi
Bhringa^a^	*Eclipta prostrata*	Shou Wu Pian
Ghana^a^	*Cyperus rotundus*	Bai Fang
Trayanthi^a^	*Gentiana kurroo*	Long Dan Xie Gan Tang

^a^ reported from the patients in this case series

An acute hepatocellular injury pattern was seen in all three patients. Two patients were jaundiced but none developed clinical signs of liver failure. Complete recovery was seen in all three cases. The patients underwent an exhaustive evaluation including viral and autoimmune serologies and imaging to rule out biliary tract or infiltrative diseases that was negative. None of the patients were re-exposed to the supplements and RUCAM scores indicated probable causality for DILI in the three patients, as detailed in Table 2.

## Discussion and conclusions

Use of ayurvedic medicine in the U.S. is more popular and extends beyond Indo-Americans as evidenced by one of the 3 patients in this cases series who is Hispanic [9]. Despite the widespread use of AM, there is a relative paucity of publications on AM-associated liver injury. With 94 patients, Philips et al. compiled the largest list of Ayurveda and herbal medicine associated with severe liver injury from a single center from southern India. Five patients including one patient who underwent liver transplantation died [27]. Other clinical literature on the subject comprises case reports, case series, and broader reviews on herb-induced liver injury [17–25, 28, 31–34]. Initial patient complaints from herbal hepatotoxicity have ranged from asymptomatic liver function test abnormalities to acute liver failure requiring transplant and/or resulting in death [27, 34]. Our report of three patients with probable AM-induced liver injury highlights the pervasive challenges of evaluating causality in herbal-medication induced liver injury. One patient took the supplement Giloy Kwath (water extraction (decoction) of herb(s)), which contained only *Tinospora cordifolia*. This herb was also present in an AM, Manjishthadi Kwatham, taken by another patient in this report. *Tinospora cordifolia* was an ingredient found in eight different formulations of AMs associated with severe liver injury [27]. For a supplement with a single herb ingredient and a uniform pattern of liver injury across multiple occurrences, such as the recent case series involving *Withania somnifera* (also known as Ashwagandha) [18], assignment of causality can be determined with greater certainty. There are other case reports of AM-associated liver injury involving a single agent; Bakuchi Powder/Babchi Seeds/Bakuchiol which consist of *Psoralea corylifolia* [21], Bristly Luffa consisting of *Luffa echinate* [35], and *Cantella asiatica* [22, 24]. However, this kind of clear association is relatively uncommon.

As in two of the patients in this series, other case reports of AM-associated liver injury involve patients taking multiple preparations that may contain numerous ingredients [20, 23, 29]. For example, one of our patients took two preparations that contained 52 and 10 individual ingredients, respectively. The large number of components is not unusual for Ayurvedic preparations. On analysis of the compendium by Philips et al. [27], 8 of the listed 27 Ayurvedic medications reported to be associated with liver injury had detailed ingredient lists available online. The mean and median number of individual herbs in these medications was 19.8 and 9, respectively, ranging from 1 to 71 ingredients per supplement (see Supplement 1). Notably, this search for ingredients may be limited by conducting it retrospectively, as ingredient listings may have since changed since their usage in the review. There were 33 total components in the products taken by patient 2 that were listed as ingredients in the preparations associated with previous cases of AM-induced liver injury; 11 such ingredients were found in the AM products taken by patient 3. While this may indicate overlap in a component or components responsible for hepatic damage, it may also simply demonstrate the ubiquity of certain ingredients in Ayurvedic medications, which adds to the challenge of attributing causality.

Another inherent challenge in evaluating toxicity related to AM and other herbal supplements is the lack of accurate data regarding quantities and dosing. For most cases, the prevailing theory of the mechanism of herbal induced liver injury is idiosyncratic and therefore, dosing is not a parameter that considered in evaluating causation [26].

To tabulate a comprehensive list of Ayurvedic compounds associated with liver injury, we performed a literature search for all articles in English discussing Ayurveda and liver injury, damage, or hepatotoxicity in humans. Articles were excluded if they detailed hepatoprotective effects. The literature review for AM-associated herbal supplement induced liver injury in humans yielded 50 results with the following distribution according to PubMed archiving: 10 case reports/series, 9 reviews of AM, 2 clinical studies, and 29 journal articles. After eliminating off-topic matches, 12 were deemed to be directly relevant for the purpose of this summary. Articles excluded were among the following categories: discussions of uses of an herb or supplement unrelated to liver injury, pharmacologic properties of a substance unrelated to liver injury, heavy metal contamination in AM, cancer unrelated to AM hepatotoxicity, and liver physiology unrelated to hepatic injury. After analyzing the compounds listed and cross referencing their constituent ingredients from available sources online (shown in Supplement 1), a number of substances were found to be common ingredients in Ayurvedic medications causing liver injury: *Phyllanthus emblica* with 12 unique occurrences, *Withania somnifera* with 11, *Zingiber officinale* with 11, and numerous others (see Supplement 2). Importantly, these compounds were not all specifically implicated in their respective studies but rather recurrently present in substances taken by patients with DILI after examination of each’s components through an online search.

With this information in mind, it is important to note the inherent limitations of this type of correlational summation. For instance, *Zingiber officinale*, though linked to 11 Ayurvedic medications [19, 27, 29] and TCM [31–33] associated with live injury, is ginger, a widely used household spice. While the ingredient could be hepatotoxic, its prevalence may also be explained by the fact that it is a common additive in Ayurvedic supplements. Similarly, *Piper nigrum* or black pepper, has 5 occurrences in medications causing liver injury, and several other compounds (see Supplement 2). This said, other commonly used products have been linked to acute liver injury, a notable example being green tea extract [36]. Until larger and more focused research is conducted, this list in Supplement 2 clearly limited in practicality by its correlational nature and should serve only as a starting ground for more focused future investigations and efforts to tabulate offending herbs or substances. Moreover, as previously mentioned, certain compounds’ ingredients were retrospectively retrieved and may not accurately reflect the exact product taken at the time of each initial case.

Like Ayurveda, TCM, which includes the use of herbs, has evolved over thousands of years. Comparatively, more research exists on Chinese herbal medications and liver injury; however, Ayurvedic and Chinese herbal medications share many ingredients. In a compilation of 57 traditional Chinese medications associated with liver injury, 18 contained similar compounds in Ayurvedic medications associated with liver injury, shown in Table 4. Moreover, an additional 5 species of the same genus as those found in the Ayurvedic medications were identified. Teschke and others have created extensive compilations of TCM herbs associated with hepatoxicity [31–34]. Assigning causality to TCM is fraught with the same challenges as AM-associated liver injury. We discovered several compounds associated with liver injury in TCM contained in the AM products taken by our three patients*: Psoralea corylifolia*, *Zingiber officinale*, *Piper nigrum*, *Cinnamomum verum*, *Cinnamomum tamala*, *Azadirachta indica*, *Eclipta prostrata, Cyperus rotundus*, and *Gentiana kurroo* [31–33]. Again, as noted above, these simply represent shared associations with liver injury in AM and TCM, which may indicate hepatotoxicity, the popularity of certain herbs in traditional medicinal preparations, or both. A 2019 review by Byeon et al. of all documented herb-induced liver injury contained substances found *Psoralea corylifolia*, which was an ingredient of Manjishthadi Kwatham taken by patient 2 in this report, to be the second most common overall precipitant from the review, with 41 occurrences [37].

The spectrum of the histological findings on liver biopsy are just as diverse and varied as the ingredients of AM that are suspected to be causative for DILI. The most common finding present on liver biopsy has been a non-specific chronic hepatitis, present in 85% of cases reported by Philips et al. [27]. However, more severe findings have been reported and include sub-massive hepatic necrosis, pan-acinar necrosis, bridging necrosis, granulomatous hepatitis, steatohepatitis, and cholestatic hepatitis [17, 19, 21, 24, 27]. Features ranging from active cholangitis, biliary proliferation, and non-specific injury typical of DILI have also been reported [21, 25, 29]. Fibrosis was present in 77% of patients in one study [27], indicating a chronic injury in the vast majority of patients, which may be attributable to long term usage of AM. The variety of histological appearances of AM related DILI presents additional challenges in routine clinical diagnosis of this etiology and further complicates attribution of the injury to one specific ingredient in AM or TCM supplements.

As AM and TCM gain wider use in the West, there is a need to develop a central registry to document herbal supplements and their constituent ingredients that have been reported to cause liver injury similar to Livertox.nih.gov, a registry of medications associated with DILI. Complicating the matter is that supplements in the United States are unregulated. The chemical composition of herbal supplements is not verified and therefore analysis is additionally challenging.

In conclusion, our case series represents three of the few documented cases of AM-induced liver injury in the United States. As Ayurvedic supplement use becomes more popular in the U.S. and globally, recognition of AM induced liver injury will become crucial. However, the complexity of supplements taken by patients make causality difficult to discern. Investigating specific ingredients in Ayurvedic medications and other herbal supplements associated with liver injury would help evaluate current correlational relationships [11]. This effort would depend on a reliable catalog of constituent ingredients, in a platform similar to that of LiverTox.gov. This would facilitate quicker recognition of offending agents when diagnosing patients with herb-induced liver injury and safer consumption of Ayurvedic as well as other complementary and alternative medicine preparations by patients.

## Supplementary Information

**Additional file 1.**

## Data Availability

All clinical data are available by contacting Dr. Tse-Ling Fong.

## Abbreviations

AM: Ayurvedic medication
USD: U.S. dollars
CAM: Complementary and alternative medicine
TCM: Traditional Chinese medicine
DILI: Drug-induced liver injury
RUCAM: Roussel Uclaf Causality Assessment Method
AP: Alkaline phosphatase
TP: Total protein
alb: Albumin
t bili: Total bilirubin
ALT: Alanine aminotransferase
AST: Aspartate aminotransferase INR international normalising ratio
ANA: Anti-nuclear antibody
SMA: Anti-smooth muscle antibody
LKM: Anti-liver kidney microsomal antibody
LDH: Lactate dehydrogenase
MRI/MRCP: Magnetic resonance imaging/magnetic resonance cholangio-pancreatography
