# Supplementary material for: Case series and review of Ayurvedic medication induced liver injury
Source: BMC Complement Med Ther. 2021 Mar 13;21:91. doi: 10.1186/s12906-021-03251-z (PMC7956115; doi:10.1186/s12906-021-03251-z)
Supplement: Supplementary file 1 — Additional file 1. [file 12906_2021_3251_MOESM1_ESM.pdf]

Supplement 1. Compilation of documented Ayurvedic medications associated with liver injury and their components

Source	# Patients	Indication	Product Name	Sanskrit Name(s)	English Name(s)	Botanical Name
Phillips et al [17]	1	Postprandial digestive supplement	Dashamoolarishtam	Bael tree (Bili, Bili Patra, Bhel)		*Aegle marmelos*
				Meloni tree		*Gmelina arborea*
				Agnimandha	Headache tree	*Premna serratifolia*
				Patala	Trumpet flower tree	*Stereospermum colais*
				Dunduka	Indian Trumpet	*Oroxylum indicum*
				Brihati	Poison berry	*Solanum anguivi*
				Kandakari	Wild eggplant	*Solanum xanthocarpum*
				Salaparni	Tick tree	*Desmodium gangeticum*
				Prsniparni	Pseudarthria	*Pseudarthria viscida*
				Gokshura	Puncture vine	*Tribulus terrestris*
				Agni	White lead wort	*Plumbago zeylanica*
				Paushkara	Elicampane	*Inula racemosa*
				Lodhra	Lodh tree	*Symplocos cochinchinensis*
				Amrita	Gulancha tinospora	*Tinospora cordifolia*
				Amalaki	Indian gooseberry	*Phyllanthus emblica*
				Duralabha	Climbing nettle	*Tragia involucrata*
				Khadira	Cutch tree	*Acacia catechu*
				Asana	Malabar kino tree	*Pterocarpus marsupium*
				Abhaya	Chebulic myrobalan	*Terminalia chebula*
				Kushta	Costus	*Saussurea lappa*
				Manjishta	Indian Madder	*Rubia cordifolia*
				Devadaru	Deodar	*Cedrus deodara*
				Vidanga	Wawrung	*Embelia ribes*
				Madhuka	Liorice	*Glycyrrhiza glabra*
				Brahmanayashtika	Beetle killer	*Clerodendrum serratum*
				Kapitha	Elephant apple	*Limonia acidissima*
				Vibhitaka	Belliric myrobalan	*Terminalia bellirica*
				Punarnava	Spreading Hogweed	*Boerhavia diffusa*
				Chavya	Wild pepper	*Piper brachystachyum*
				Jatamansi	Spikenard	*Nardostachys jatamansi*
				Priyangua	Calumpang Nut Tree	*Callicarpa macrophylla*
				Sariba	Indian sarasaparilla	*Hemidesmus indicus*
				Kalonji	Fennel flower	*Nigella sativa*
				Trivrut	Indian Jalap	*Operculina turpethum*
				Renuka	Orange pepper tree	*Piper aurantiacum*
				Rasna	Java galangal	*Alpinia calcarata*
				Pippali	Long pepper	*Piper longum*
				Kramuka	Areca-nut palm	*Areca catechu*
				Sathi	Aromatic Ginger	*Kaempferia galanga*
				Haridra	Turmeric	*Curcuma longa*
				Satapushpa	Dill Weed	*Anethum graveolens*
				Padmaka	Himalayan wild cherry	*Prunus cerasoides*
				Padmaka	Himalayan wild cherry	*Mesua ferrea*
				Musta	Coco grass	*Cyperus rotundus*
				Indrayava / Kutaja	Kurchi	*Holarrhena antidysenterica*
				Srngi	Crab's claw	*Pistacia integerrima*
				Jivaka	Jeevak	*Malaxis acuminata*
				Rishabaka	Rishabak	*Malaxis muscifera*
				Meda	Meda	*Polygonatum verticillatum*
				Mahameda	Mahameda	*Polygonatum cirrhifolium*
				Draksha	Grape wine	*Vitis vinifera*
				Kakkola	Star anise	*Illicium verum*
				Hribaram	White cus cus grass	*Coleus vettiveroides*
				Chandana	White sandal tree	*Santalum album*
				Jati	Mace tree	*Myristica fragrans*
				Devapushpa	Clove tree	*Syzygium aromaticum*
				Twak	Cinnamon Tree	*Cinnamomum verum*
				Ela	Cardamom	*Elettaria cardamomum*
				Tamalapattra	Indian Bay Leaf	*Cinnamomum tamala*
				Agakesara / Nagapushpa	Iron wood tree	*Mesua ferrea*
				Pippali	Long pepper	*Piper longum*
				Dhataki	Fire flame bush	*Woodfordia fruticosa*
				Guda	Sugar cane	*Saccharum officinarum*
				Makshika	Honey	Honey
Björnsson et al [18]	3		NOW Ashwagandha	Ashwagandha Extract	Indian Ginseng, Poison Gooseberry, and Winter Cherry	*Withania somniferia*
				Cellulose		
				Rice flour		
				Magnesium stearate		
	1		Nature's Way Ashwagandha	Ashwagandha (root)		*Withania somniferia*
				Plant derived capsule		
				Rice powder		
				Magnesium stearate		
	1		N/A	Ashwagandha*	Indian Ginseng, Poison Gooseberry, and Winter Cherry	*Withania somniferia*
Douros et al [19]	1	unknown, used tea "several times"	Ayurvedic Tea		Licorice	*Glycyrrhiza glabra*
					Ginger	*Zingiber officinale*
					Cardamom	*multiple potential*
					Cinnamon	*multiple potential*
Teschke and Bahre [20]	1	Vitiligo	N/A	Bakuchi	Babchi Seeds	*Psoralea corylifolia*
				Khadin		*Acacia catechu*
				Brahmi		*Bacopa monnieri*
				Usheer		*Vetivexia zizaniodis*
Smith and MacDonald [21]	1	Vitiligo		Bakuchi	Babchi Seeds	*Psoralea corylifolia*
Dantuluri et al [22]	1	Acne		Gotu Kola		*Cantella asiatica*

Tremlett et al [23]	1	Multiple Sclerosis	AP Mag Capsules (Ayush Herbs)	Bael tree (Bili, Bhel)		*Aegle marmelos*
				Nim Patra / Neem Patra	Indian Lilac	*Azadirachta indica*
				Chutro	Indian barberry	*Berberis aristata*
				Vidanga	False Black Pepper	*Embelia ribes*
				Indrayava	Kutaja seeds	*Holarrhena antidysenterica*
				Karela	Bitter Melon	*Momordica charantia*
				Tulsi		*Ocimum sanctum*
				Pippali	Long pepper	*Piper longum*
				-	Vegetarian Capsule	-
				-	Magnesium stearate	-
				-	Silicon Dioxide	-
			Ashwagandha Capsules (Ayush Herbs)	Ashwagandha	Indian Ginseng, Poison Gooseberry, and Winter Cherry	*Withania somnifera*
				-	Providing Withanoloides	-
				-	Hypromellose	-
				-	Titanium Dioxide	-
				-	Caramel Colour	-
				-	Silicon Dioxide	-
				-	Magnesium stearate	-
			Livit-2 (Ayush Herbs)	Sarapunkha		*Tephrosia purpurea*
				Bhuamlaki		*Phyllanthus amarus*
				Kiratatikta / Chirayata		*Swertic chirata*
				Bhringraj		*Eclipta alba*
				Arka		*Calotropis giganttea*
				Moolaka / Mulaka		*Raphanus sativa*
				Daruharidra		*Berberis aristata*
				Arjuna/Arjun		*Terminalia arjuna*
				Vibhitaka		*Belleric myrobalan*
				Haritaki		*Terminalia chebula*
				Amla	AMALOXY ™	*Emblica officinalis*
				Kakamachi		*Solanum nigum*
				Kalamegha		*Andrographis paniculata*
				Guduchi / Giloy		*Tinospora cardifolia*
				Katuki		*Picrorhiza kurroa*
				Punarnava		*Boerhavia diffusa*
				-	Calcium Carbonate	-
				-	Stearic Acid	-
				-	Croscarmellose sodium	-
				-	Microcrystalline Cellulose	-
				-	Magnesium stearate	-
				-	Silicon Dioxide	-
				-	Food Glaze Coating	-
			Thorne research-L-Glutamine Capsules		L-glutamine	
			Thorne Research MediClear Supplement		*mutlivitamin*	
Jorge and Jorge [24]	3	Weight Loss	N/A	Gotu Kola		*Cantella asiatica*
Shiyovich et al [25]	1	Type 2 diabetes mellitus	Gymnema sylvestre			*Gymnema sylvestre*
Phillips et al [27]	1	arthritis	yog sallaki			*boswelllia serrata*
	1	arthritis	bonsil	abha guggal		
				laxadi (lakshadi) guggal		*laccifer lacca*
						cissus quadrangularis
						terminalia arjuna
						*withania somifera**
						grewia populifolia
						commiphora mukul
				madhumalini vasant		
				kachur		*curcuma zedoaria*
				priyangu		*aglaia elaeagnoidea*
				kukkuta anda	egg yolk	
				kishore (kaishore) guggal		*emblica officinalis*
						terminlia bellirica
						terminlia chebula
						commiphora mukul
						tinospora cordifolia
						zingiber offininale
						piper nigrum
						piper longum
						embelia ribes
						baliospermum montanum
						ipomoea turpethum
				praval bhasma	calcium	
				kukutandatwak bhasma	calcium	
				pushpadhanva ras		
				ras sindoor	shudda parada (Hg)	
					shudda gandhaka (S)	*cinnamomum zeylanicum*
						elettaria cardamomum
						cinnamomum tamala
						mesua ferrea
						tinospora cardifolia
						terminalia bellicrica
						phyllanthus emblica
						zingiber officinale
						eclipta aba
					vatankura	*ficus benghalensis*
				nag bhasma	amorphous lead (Pb)	
				lauh bhasma	iron (Fe)	
				abhrak bhasma	mica	
				bang bhasma	tin (Sn)	

1	arthritis	ostina forte	asthishrinkhala		*cissus quadrangularis*
			arjuna		*terminalia arjuna*
			amalaki		*emblica officinalis*
			abhraka bhasma	mica	
			lakshadi guggulu		*laccifer iacca*
					cissus quadrangularis
					terminalia arjuna
					withania sommifera
					grewia populifolia
					commiphora mukul
1	arthritis	swarna guggulu	swarna bhasma	gold (Au)	
			ashvagandha	withania sommifera	*withania sommifera*
			kumkuma	Tumeric	
					Crocus sativus
1	cardiac benefits	bitana forte			
1	-	bacfo joint care			
1	-	ikshwadi			
1	-	sankara bati			
1	-	hriden plus			
1	-	vasaguluchyadi	vasa		*adhatoda vasica*
			guduchi-giloy		*tinospora cordifola*
			triphala-haritaki		*terminalia chebula*
			vibhitaki		*terminalia bellirica*
			amalaki		*emblica officinalis*
			katvi		*picrorrhiza kurroa*
			bhunimba		*andrographis paniculata*
			nimba-neem		*azadirachta indica*
1	-	prameoushadi			
1	arthritis	orthoherb	eranda	castor bean	*ricinus communis*
			vasa		*adhatoda vasica*
			vilvam	bilva fruit	*aegle marmelos*
			sahacharam		*barleria prionitis*
			bala		*sida corifolia*
			nimba		*azadirachta indica*
			dusparsha		*tragia involucrata*
			gunja		
			pata		
			maricham		
			thwak		
			jati phalam		
			lavangam		
			amalaki		
			manjishta		
			nirgundi		
			punarnava		
			guggulu		
			chitharakam		
			katakah		
			gokshurah		
			shatavari		
			pashnabheda		
1	-	Livomyn			
1	-	ayamodaka sathaal			
1	-	kaisoragulu vatika			
1	-	najati powder			
1	-	vilwadi gulika			
1	-	testes siccata			
1	-	oleum jeconis			
1	-	arethaki lehyam			
1	-	drakhati kath			
1	-	G H evandam			
1	-	gasna syrup			
1	-	noni			

1	-	rasn(a) sallaki	rasna		*alpinia galanga*
			sallaki		*boswellia serrata*
			brihat panchamoola		
			bilva		*aegle marmelos*
			agnimantha		*premna integrofolia*
			shyonaka		*oroxylum indicum*
			patala		*stereospermum suaveolens*
			gambhari		*gmelina arborea*
			bilva		*aegle marmelos*
			agnimantha		*premna mucronata*
					premna integrifolia
			shyonaka		*oroxylum indicum*
			patala		*stereospermum suaveolens*
			gambhari		*gmelina arborea*
			laghu panchamoola		
			brihati		*solanum indicum*
			kantakari		*solanum xanthocarpum*
			shalaparni		*desmodium gangeticum*
			prishnaparni		*uraria picta*
			gokshura		*trbulus terrestris*
			punarnava		*boerhavia diffusa*
			saunth	ginger	*zingiber officinale*
			gokharu		*tribulus terrestris*
			bala		*sida corifolia*
			malkangni		*celastrus panculatus*
			erand		*ricinus communis*
			giloy		*tinospora cordifolia*
			kuchla		*strychnos nuxvomica*
			ajwain	carom seeds	*trachyspermum ammi*
			khurasani ajwain		*hyoscyamus niger*
			jatamansi		*nardostachys jatamansi*
			chob chini		*smilax china*
			ashwagandha		*withania somnifera*
			mahayograj (guggul)		
				ginger	*zingiber officinale*
				long pepper	*piper longus*
			chavya		*piper cubeba*
			pippalimoola		*piper longus*
			hingu		*asa foetia*
			chitraka		*plumbago zeylanica*
			ajamoda		*trachyspermum roxburghianum*
			sarshapa		
			shwetajeeraka		*cuminum cyminum*
			krishnajeeraka		*carum carvi*
			renuka		*vitex agnus castus*
			indrayava		*hlarrhena antidysenterica*
			patha		*cyclea peltata/cissampelos pariera*
			videnga		*embelia ribes*
			gajapippali		*piper chaba*
			katuka		*picrorrhiza kurroa*
			ativisha		*aconitum heterophyllum*
			bharngi		*clerodendro serratum*
			vacha		*acorus calamus*
			murva		*marsdenia tenacissma*
			triphala (haritaki-vibhitaki-amla		*embelia officialis*
					terminalia chebula
					terminalia bellirica
			shuddha guggulu		*commiphora mukul*
			vanga bhasma		tin
			roupya bhasma		silver
			naga bhasma		lead
			loha bhasma		iron
			abharaka bhasma		mica
			mandura bhasma		iron
			rasasindhura		
			guggul	guggul plant	*commiphora mukul*
			shilajeet (shilajit)	fulvic acid	
			swarnmakshik	copper	
				iron	
			kapardik	cypraea moneta	cowries
			shankha bhasma	conch shell	
1	-	ginseng		ginseng	*Panax ginseng*
1	-	unknown	-	-	-

Dalal et al [29]	1	*Not discussed*	Punarnava mandur	Punarnava		*Boerhavia diffusa*
				Nishoth roots		*Operculina turpethum*
				Sonth	Ginger Root	*Zingiber officinale*
				Kali Mirch	Black Pepper	*Piper nigrum*
				Pippali	Long Pepper	*Piper longum*
				Vaividang	False Black Pepper	*Embelia ribes*
				Devdaru	Himalayan Cedar	*Cedrus deodara*
				Chitrak root	-	*Plumbago zeylanica*
				Kushta	Indian Costus Root	*Saussurea lappa*
				Haldi	Turmeric	*Curcuma longa*
				Haritaki	-	*Terminalia chebula*
				Bibhitaki	-	*Terminalia bellirica*
				Amla	Indian Gooseberry	*Emblica officinalis*
				Danti roots	-	*Baliospermum montanum*
				Chavya	Java Long Pepper	*Piper chaba*
				Indrayava		*Holarrhena antidysenterica*
				Kutki	-	*Picrorhiza kurroa*
					Long Pepper Roots	*Piper longum*
				Mustak	Nut Grass	*Cyperus rotundus*
				Kakra Singhi	Crab's claw	*Pistacia integerrima*
				Kala Jeera	Caraway	*Carum carvi*
				Carom seeds		*Trachyspermum ammi*
				Ajwain	Carom Seeds	*Trachyspermum ammi*
				Kayaphal		
				Mandur bhasma	Ferric Oxide	
					Triphala decoction	*Amalaki (Emblica officinalis)*<br>*Bibhitaki, (Terminalia belerica)*<br>*Haritaki (Terminalia chebula)*
					Cow's urine	
					Aloe vera	
				Cow's urine		
		Not discussed	Kanchnar guggulu	Kanchanar bark		*Bauhinia variegata*
				Guggulu resin		*Commiphora mukul*
				Amalaki fruit		*Emblica officinalis*
				Babhitaki fruit		*Terminalia belerica*
				Sonth	Ginger root	*Zingiber officinale*
				Kali Mirch	Black Pepper fruit	*Piper nigrum*
				Pippali fruit	Long Pepper	*Piper longum*
				Varuna bark		*Crataeva nurvala*
					Cinnamon bark	*Cinnamomum verum*
					Cardamom seed	*Elettaria cardamomum*
				Tamalpatra leaf		*Cinnamomum tamala*
Giri et al [35]	1	Digestion and bloating	Dried fruits of Luffa echinata soaked in water			*Lucha echinata*
Gunturu et al [37]	1	Diabetes	Jambrulin (Unjha Ayurvedic Pharmacy)	Jamungiri extract	*unknown*	
				Mamejava extract		*Enicostemma littorale*
				Bael tree (Bili, Bili Patra, Bhel)		*Aegle marmelos*
				Tribang Bhasma	Tin	-
					Lead	-
					Zinc	-
				Gudmar		*Gymnema sylvestre*
				Nim Patra		*Azadirachta indica*
				Refined Shilajit		*Shilajit/Mineral pitch/mumijo*

Supplement 2. Number of occurrences of substances recurrently associated with AM-associated injury across current literature

**Components**	**# of Occurences**
Phyllanthus emblica	12
Withania Somnifera	11
Zingiber officinale	11
Terminalia bellirica	8
Tinospora cordifolia	8
Terminalia chebula	7
Piper longum	7
Embelia ribes	6
Azadirachta indica	5
Commiphora wightii	5
Piper nigrum	5
Elettaria Cardamomum	4
Cinnamomum verum	4
Boehavia diffusa	4
Centella asiatica	4
Psoralea corylifolia	3
Boswellia serrata	3
Cissus quadrangularis	3
Terminalia arjuna	3
Iron	3
Mica	3
Commiphora mukul	3
Curcuma longa	3
Andrographis paniculata	3
Aegle marmelos	3
Tribulus terrestris	3
Cyclea peltata	3
Bauhinia variegata	2
Laccifer lacca	2
Grewia Populifolia	2
Baliospermum montanum	2
Ipomoea turpethum	2
Calcium	2
Mercury	2
Cinnamomum tamala	2
Eclipta alba	2
Ficus benghalensis	2
Lead	2
Tin	2
Adhatoda vasica	2
Picrorrhiza kurroa	2
Ricinus communis	2
Sida corifolia	2
Tragia involucrata	2
Rubia cordifolia	2
Tephrosia Purpurea	2
Cedrus Deodara	2
Stereospermum suaveolens	2
Plumbago zeylanica	2
Holarrhena antidysenterica	2
Clerodendrum serratum	2
Acorus calamus	2
Marsdenia tenacissima	2
Cassia fistula	2
Holarrhena pubescens	2
Trichosanthes cucumerina	2
